# Diagnosis of Evans syndrome

**DOI:** 10.11604/pamj.2021.38.314.22410

**Published:** 2021-03-29

**Authors:** Andreas Angelopoulos, George Kirkilesis, Kiriaki Kakavia, Paraskevi Papanikolaou

**Affiliations:** 1 Cardiology Department, 417 NIMTS Hospital, Athens, Greece,; 2 Department of Surgery, General Hospital of Pyrgos, Ilia, Greece,; 3 Department of Vascular Surgery, Laikon General Hospital, Athens, Greece

**Keywords:** Evans syndrome, thrombocytopenia, anemia, hemolysis

## Abstract

This manuscript concerns the case of a patient hospitalized and diagnosed with Evans syndrome. She was hospitalized with signs of thrombocytopenia induced purpura, petechiae, ecchymosis and anemia. She was successfully treated with corticoids and blood transfusions. Our purpose is to explain her clinical presentation and the exams, we used in order to make the diagnosis of Evans syndrome, which requires great suspicion. Moreover, other diseases causing hemolytic anemia and thrombocytopenia must be excluded. We used laboratory tests (blood samples, Coombs examination and virologic test). Bone marrow examination took place twice. Evans syndrome is an autoimmune disease which is characterized by the coexistence of hemolytic anemia and immune-mediated thrombocytopenia. There is no typical clinical presentation. Its etiology is unknown and its therapy is generally poor. Diagnosis of Evans syndrome is very difficult and requires the exclusion of other diseases causing anemia and thrombocytopenia.

## Introduction

Evans syndrome was first described in 1951 and consist of a rare clinical condition combining autoimmune hemolytic anemia (immediate Coombs positive) as well as thrombocytopenia [autoimmune hemolytic anemia (AIHA) and Idiopathic thrombocytopenic purpura (ITP) coexistence] without still knowing its etiology [1]. The frequency of the syndrome is also unknown. While in children it is more common among boys rather than girls at a ratio of 1.4: 1, in adults women seem to be those who suffer the most [1,2]. It is listed as a rare disease from Office of Rare Diseases (ORD) of National Institute of Health (NIH), meaning that it is common in less than 200,000 people of the U.S population. Its typical clinical course is characterized by periods of exacerbation and recession. Correspondence to treatment has been poor so far [3,4]. First type of therapy is the glucocorticoids (prednisolone, methylprednisolone) as well as intravenous immune globulin (IVIG) used in ITP [5]. A new approach introducing rituximab has shown good results mostly in those cases which correspond to corticoids [6,7]. Splenectomy also appears to be a timeless solution entailing many relapses though [8].

## Patient and observation

**Figure 1:**
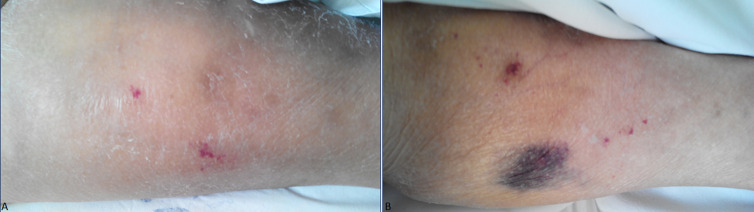
appearance of ecchymosis and petechiae all over the body and limbs of the patient

We are going to present the case of an 83-year-old woman, who was taken into the emergency department of Pyrgos General Hospital in February 2015, because of diffusible ecchymosis and petechiae all over her body and limbs. The ecchymosis was diffusible and especially umbilical of a diameter up to 6-7cm (Figure 1). From the clinical examination, there were normal respiratory whispering and softly pushed painless abdomen without a groping liver or spleen. According to her medical history, she presented atrial fibrillation and she was taking acenocoumarol. The laboratory results of the patients were the following, HCT 23.6%, HGB 7.4 gr/dl, RBC 2.36 K/μl, WBC 5400/μl (NEUT 47.8% LYM 26.1% MONO 25% EOS 0.9% BASO 0.2%) PLT 61000/μl. In biochemical testing dextrose 84mg/dl urine 32 mg/dl creatinine 0.7 mg/dl SGOT 19 IU/L SGPT 8 IU/L BIL 1,1 mg/dl γ-GT 19 IU/L, total albumins 6.9 gr/dl albumin 3.9 gr/dl CRP 1.1 mg/dl total globulins 3.0 gr/dl. The results of coagulation test were the following, Prothrombin Time 15.28 sec, Partial Thromboplastin Time 26.4 sec and INR 1.52.

As for the rest of the testing, peripheral blood smear was examined and nothing pathological was found. Moreover, bone marrow examination, taking place in private laboratory, was normal. Immediate Coombs was positive and the virologic test, including HBsAG, anti -HCV antibodies, anti EBV (IgM, IgG) antibodies, anti CMV (IgM, IgG) antibodies, was negative. A collagenous testing wasn't carried out. The patient was treated with transfusions of red blood cells concentrates and fresh frozen plasma (FFP), as well as 120 mg/day intravenous methylprednisolone. Her clinical presentation improved with an ascent of the hematocrit to 33%. However, her blood cells remain at the same level. The patient was nursed for 17 days in total. A second hospitalization of the patient followed the next month with a thrombocytopenia relapse. During this hospitalization, patient was treated with prednisolone at the dose of 1.5 mg/kg/day. Patient presented great correspondence to treatment as her platelets ascended to 113 K/ml. This presentation is most consistent with Evans syndrome.

## Discussion

Evans syndrome diagnosis requires a high indicator of suspicion as well as exclusion of many other disturbances causing autoimmune hemolytic anemia and thrombocytopenia [9]. The etiology and pathogenesis of the syndrome are not fully clarified [10]. To make a diagnosis, more diseases causing cytopenia should be excluded, like the systematic erythematous lupus, the thrombotic thrombopenic purpura (TTP), the marrow dysplastic, the lymphohyperplastic syndromes as well as the viral infectious diseases [11,12]. The patient didn´t fulfill any of the clinical criteria of systematic erythematous lupus as defined by the Contemporary American College of Rheumatology. There should also be made a differential diagnosis from thrombotic thrombopenic purpura. The clinical five symptoms are: thrombocytopenia, microvasculopathic hemolytic anemia, kidney disease, fever and neurological deficiencies appearance. However, the clinical image appears with all symptoms in less than 35% of the patients. The patient didn´t feature any other symptom except for thrombocytopenia and autoimmune hemolytic anemia. Furthermore, no schistocytes, which are distinctive of Thrombotic Thrombocytopenic Purpura (TTP), were found in the peripheral blood smear although it was examined twice. Moreover, as the marrow testing didn´t prove any pathological findings, both marrow dysplastic and lymphohyperplastic diseases were eliminated.

## Conclusion

Evans syndrome may appear having autoimmune thrombocytopenia as a first symptom. Needless to say, diagnosis is hard to make and normally requires that all the other systematic diseases reported be eliminated by means of clinical or laboratory testing [13]. Additionally, this type of syndrome is hard to correspond to the various treatments suggested. In conclusion, its diagnosis and mainly its treatment consist of a great challenge for the whole medical community.
